# Dynamics of *pfcrt *alleles CVMNK and CVIET in chloroquine-treated Sudanese patients infected with *Plasmodium falciparum*

**DOI:** 10.1186/1475-2875-9-74

**Published:** 2010-03-12

**Authors:** Nahla B Gadalla, Salah Eldin Elzaki, Ebtihal Mukhtar, David C Warhurst, Badria El-Sayed, Colin J Sutherland

**Affiliations:** 1 Department of Infectious and Tropical Diseases, London School of Hygiene and Tropical Medicine, UK; 2 Department of Epidemiology, Tropical Medicine Research Institute, Khartoum, Sudan

## Abstract

**Background:**

Parasite resistance to the anti-malarial drug chloroquine is common in eastern Sudan. Dynamic within-host changes in the relative abundance of both sensitive and resistant *Plasmodium falciparum *parasites were examined in a cohort of chloroquine-treated patients presenting with uncomplicated falciparum malaria, using a novel allele-specific quantitative approach.

**Methods:**

Treatment outcomes were determined for 93 patients of all ages in a per protocol cohort using a modified 14-day WHO protocol. Parasite DNA samples at days 0, 1, 2, 3, 7 and 14 following treatment were analysed using real-time quantitative PCR methods that distinguished resistant and sensitive genotypes at amino acids 72 - 76 of the *pfcrt *locus.

**Results:**

Chloroquine treatment was not efficacious, and of 93 assessable patients, only 10 individuals (10.7%; 95% C.I. 4.34 - 17.2%) enjoyed an adequate clinical and parasitological response. Resistant parasites with the haplotype CVIET at codons 72-76 of the *pfcrt *locus were dominant in the starting population. Chloroquine sensitive parasites with the haplotype CVMNK were detected in 19 individuals prior to treatment (20.43%; 95% C.I. 5.14 - 18.5%). In these patients, CQ treatment rapidly selected CVIET parasites, and this haplotype overwhelmingly dominated the parasite population in each individual by day 2 after treatment.

**Conclusions:**

Such rapid intra-host selection of particular genotypes after the introduction of drug will cause frequent misidentification of parasite genotypes present in the starting population. This will have a potentially serious confounding effect on clinical trials which employ PCR-corrected estimates of treatment failure, as resistant parasites below the detection threshold in the pre-treatment sample can be erroneously classified as "new" infections during follow-up, over-estimating drug efficacy.

## Background

The malaria parasite *Plasmodium falciparum *causes considerable morbidity and mortality in the tropics, exacerbated in the last decade of the 20^th ^century by widespread drug resistance in endemic areas [1]. Resistance to chloroquine, the cheapest and most widely available anti-malarial, has reached significantly high levels leading to replacement with artemisinin-based combination therapy (ACT) in many malaria-endemic countries [2]. Genetically, chloroquine resistance (CQR) has been linked to 15 polymorphisms in the *pfcrt *gene from different parts of the world [3-6], as well as mutations in *pfmdr1 *[7,8]. Of particular interest are mutations in the region of *pfcrt *encompassing codons 72 - 76. Substitutions in the wild type allele, encoding CVMNK, give rise to several resistant variants, of which the most common are CV**IET **in South-East Asia and Africa and **S**VMN**T**, which has been reported in South America [3] and Asia [9], but rarely in Africa [10]. The single codon change at position 76, found in all natural CQR isolates to date, is widely accepted as the best molecular marker for field surveys of CQR *P. falciparum*. Prolonged use of chloroquine monotherapy has imposed high selection pressure, leading to a substantial increase in the prevalence of this marker in parasite populations worldwide.

As CQ is replaced by new combination therapies in many endemic countries, it is important from an epidemiological point of view to monitor changes in frequency at all loci associated with CQR as the effect of decreased chloroquine pressure on the parasite population is felt. Longitudinal measurements of the prevalence of CQR loci in a highly seasonal setting suggest that sudden reduction in the intensity of CQ drug pressure after the malaria transmission season and through the dry (non-transmission) season can lead to significantly lower prevalence of CQR parasites [11]. This supports other studies that have found evidence that CQR parasites are intrinsically less fit than wild-type parasites in the absence of CQ drug pressure [12,13]. Therefore, the effects of the introduction of ACT in Sudan may be dramatic, and could rapidly reduce the prevalence of  CQR parasites, as was seen in Malawi after removal of CQ from the health system [14-16]. This selection may, however, cause an increased parasite "tolerance" for ACT [17,18].

Recently developed quantitative PCR (qPCR) techniques employing dual-labelled probes allow for simultaneous allelic discrimination of two to six loci. This technique has been found specific and sensitive in the detection of drug resistance alleles of *P. falciparum *[9,19,20]. Moreover the ability to quantify the abundance of each allele in mixed genotype infections allows detailed study of the dynamics of competition between parasites with and without CQR alleles.

In this study, allele-specific quantification is employed to study the abundance of parasites with wild-type and CQ resistant genotypes at positions 72 - 76 of *pfcrt *in Sudanese malaria patients before and after treatment with CQ. Samples were collected during a clinical study in 2004, immediately before the deployment of ACT in Sudan, and provide detailed evidence of the intra-host dynamics of parasites under CQ treatment in this highly seasonal transmission setting prior to a dramatic change in drug pressure.

## Methods

### *In vivo *study

#### Sample size

The sample size was calculated using EPINFO software on the basis of the expected proportion of *pfcrt*-76T in the population of 70% in eastern Sudan [12,21]. At a confidence level of 95%, a study with 100 patients was predicted to give 80% power to detect a difference in treatment outcome between infections harbouring parasites with resistant versus sensitive *pfcrt *alleles.

#### Study area

Eastern Sudan is characterized by a markedly seasonal, short malaria transmission season lasting a few weeks every year. CQR has been reported since the mid 1980s [22] and further studies indicate that this phenotype is well established in the area [23,24]. Levels of CQR are reported to be higher than other parts of the country [21].

#### Patient recruitment

Symptomatic patients in Asar village were recruited into a community-based CQ anti-malarial clinical study. The WHO protocol for monitoring *in vivo *anti-malarial efficacy recommends 28-days of follow-up [25], however a 14-day protocol was followed as failure rates were anticipated to be high. The study included all age groups, as adults are equally susceptible to infection in this low endemicity setting.

Patients diagnosed with uncomplicated *P. falciparum *mono-infection and who had axillary temperatures of 37.5°C or above were eligible to participate in the study. Patients meeting all enrolment criteria as per established study protocols [25], who did not have any other disease, and who provided their written informed consent were recruited into the trial. A standard three-day dose of chloroquine (25 mg/kg, IDA, Netherlands) was provided by the study team under observation. Patients were observed for 30 minutes after treatment. If vomiting occurred within 30 minutes of treatment, a full dose was repeated. If vomiting occurred between 31 min and one hour after treatment, half a dose was repeated. Patients with persistent vomiting were referred to Gedaref Teaching Hospital for appropriate treatment and withdrawn from the study. Pregnant women were referred to Gedaref Teaching Hospital. Patients who refused to participate in the study or decided to withdraw were provided with a regimen of three daily doses of artesunate plus a single dose of sulphadoxine-pyrimethamine on day 0, as recommended by the national malaria control programme.

Once enrolled the patient was given a follow-up schedule for attending the clinic, on days 1, 2, 3, 7 and 14 after treatment. Thick and thin blood films were prepared and blood drops on filter paper were collected on each follow-up day. Parasite count was carried out against 200 WBCs and parasite densities were calculated assuming a WBC count of 6,000/μl.

Patients and their guardians were also advised to return to the clinic if they developed any symptoms on any other day during the study. Patients with recurrent malaria symptoms from day 3 or later during follow-up were withdrawn from the study and treated with artesunate plus sulphadoxine-pyrimethamine as above. A number of patients with low density asymptomatic parasitaemia at day 3 were not identified as parasite positive in the field clinic during follow-up and were seen again on day 7. Subsequent, definitive microscopy found day 3 parasites in blood films from these individuals. Therefore, these patients were retrospectively classified as "early treatment failure" cases, despite the additional collection of a day 7 sample. All samples collected contributed to the genetic analysis. At no stage did the study objectives override the patients' health and safety.

#### Sample collection and storage

Blood films for parasite count were collected and examined by microscopists on days 0, 1, 2, 3, 7 and 14 or on any other day if required, a parallel drop of blood was collected onto filter paper for DNA analysis.

#### Study end points

The *in vivo *study end points were taken directly from the WHO 28-day protocol, adapted for 14 days follow-up. The clinical endpoints were:

1) early treatment failure (ETF; parasitaemia on Day 2 higher than Day 0, parasitaemia on Day 3 with axillary temperature ≥ 37.5°C; or persistent parasite density greater than 25% of the presentation density on days 1 or 2).

2) late clinical failure (LCF; recurrent symptoms with parasitaemia on any day from 4 to 14)

3) late parasitological failure (axillary temperature ≥ 37°C or history of fever or asymptomatic parasitaemia on day 7 to 14)

4) adequate clinical and parasitological response (ACPR; parasitaemia absent days 3 to 14).

### Quantitative real-time PCR (qPCR)

DNA was extracted from blood-spots as in previous studies [18], to yield approximately 100 μl of supernatant containing DNA, which was stored at -20°C.

Double-labelled probes crt76-CVMNK, crt76-CVIET and crt76-SVMNT were designed to detect the three most common haplotypes at codons 72 to 76 of the *pfcrt *gene. Each probe was dual-labelled with a reporter dye at the 5' end and a quencher moiety at the 3' end (Table 1). Previous studies described the use of this assay for *pfcrt *genotyping of imported cases of falciparum malaria in the UK [9,26].

**Table 1:** Double-labelled probes for *pfcrt *genotyping.

Probe	5' Fluorophore	Sequence	3' Quencher
crt76-CVMNK	FAM	TGT GTA ATG AAT AAA ATT TTT GCT AA	BHQ1

crt76-CVIET*	JOE	TGT GTA AT**T G**A**A **A**C**A ATT TTT GCT AA	BHQ 2

crt76-**S**VMNT*	ROX	**A**GT GTA ATG AAT A**C**A ATT TTT GCT AA	BHQ1

*Amino acid and nucleotide positions deviating from the wild-type are shown in bold.

A 25 μl reaction volume contained 0.2 μM dNTPs, 0.3 μM of each forward CRTD1 and reverse CRTD2 primer (Djimde, Doumbo et al. 2001), 0.1 μM of each probe (MWG-Biotech), 1× (NH_4_)_2_SO_4 _reaction buffer, 3 mM MgCl_2 _and 1 unit of BIOTAQ™ DNA Polymerase (Bioline).

DNA from 3D7, Dd2 and 7G8 clones, representing the three most widespread *pfcrt *alleles CVMNK, CVIET and SVMNT at codons 72-76 respectively, were obtained from the Malaria Research & Reference Reagent Resource (MR4, Manassas, Vermont, USA) and employed to establish the technique and determine the sensitivity and specificity of the assay. Genotypes were identified by allelic discrimination analysis based on which of the reporter dye signals accumulated in each sample. Once established the technique was employed in field samples to identify the corresponding alleles. 5 μl of DNA from each sample was added to the reaction components and analysed as described above.

For qualitative experiments, a single multiplex PCR reaction was run for each sample on each day of follow-up. Relatively conservative thresholds were set manually, by reference to the appropriate positive and negative controls on each run.

For quantitative experiments, multiplex qPCR reactions were run in triplicate for each sample and each control. The increased precision afforded in these experiments facilitated the setting of less conservative thresholds, by reference to control DNA run in triplicate. Relative quantitation was performed using a variation of the delta-delta C_T _(cycle threshold) method of Price *et al *[27]. Briefly, DNA from the day 0 sample of patient #57, which carried both CVMNK and CVIET *pfcrt *alleles at relatively high abundance (mean C_T _for CVMNK and CVIET of 27.12 and 26.26, respectively) was selected as the calibrator and arbitrarily assigned a relative value of 1.00. Relative abundance is then calculated as 2^-ΔΔC^T where ΔΔC_T _is equal to the mean over three replicates of (C_T _^CVMNK ^- C_T_^CVIET^) for the test sample minus the mean of (C_T _^CVMNK ^- C_T_^CVIET^) for the calibrator sample (patient #57 day 0). All DNA samples from day 0, 1, 2 and 3 were then evaluated for these 17 individuals and plotted on an arbitrary logarithmic scale from 0.0001 (CVMNK only) to 100.00 (CVIET only). This relative allele abundance measure is a ratio of the difference between the fluorophore signal for each allele in the test sample, and that for the calibrator sample, and thus has no units.

### Sequencing

Forty field samples were randomly selected for direct sequencing to evaluate the specificity of the real-time PCR technique. Parasite DNA was amplified using primers CRTP1 [4] and P6 5'-TCATTTTGGCCTTCATAGGTCT-3' in 25 of the samples while 15 samples failed to produce a PCR product. These samples were subjected to a hemi-nested PCR by CRTP1 and P8 5'-GCCTGTATGCTTTTCAAACATG-3' in the first round and CRTP1 and P6 in the second round of PCR. PCR products were purified using the QIAquick PCR Purification Kit (Qiagen, U.K). Sequencing was performed using the BigDye^® ^Terminator v3.1 Cycle Sequencing Kit (Applied Biosystems, USA) as recommended by the manufacture and analysed by ABI 7300. Sequences were viewed and edited in Chromas version 2.31 and aligned in Bioedit sequence analysis software.

### Data analysis

*In vivo* data were entered and analysed according to the 2003 WHO protocol for areas of low transmission, with 14 days follow-up [28]. Molecular data were analysed using Stata 10 software package. Odds ratios were tested for significance using Fisher's exact test.

### Ethical considerations

Ethical clearance was obtained from the National Ethical Review Committee of the Federal Ministry of Health, Sudan, the WHO/TDR Ethical Review Committee and London School of Hygiene & Tropical Medicine Ethical Review Committee.

## Results

### Study population

One hundred and five patients from Asar were recruited to the study. Baseline characteristics for the study population are shown in Table 2.

**Table 2:** Base line characteristics of the study population.

Mean age yrs (min - max)	18.3	(5 - 76)
Female n (%)	54	(57.6)
Male n (%)	39	(42.4)
Mean temp °C day 0 (min - max)	37.8	(37.5 - 40.9)
Geometric mean parasitaemia day 0; parasites/μl (95% C. I.)	2,305	(1,683 - 3,156)
**Total completed follow-up**	**93**	

### Efficacy of chloroquine in *P. falciparum *in Gedaref

105 patients were originally enrolled, twelve patients either withdrew consent or were lost to follow-up, leaving 93 in our per protocol analysis. Ninety-three (88.6%) patients completed the CQ treatment course and reached one of the study end-points. Four patients (4.3%) with persisting symptoms and parasitaemia were withdrawn and administered rescue treatment prior to day 3. Ten patients (10.8%) exhibited adequate clinical and parasitological response (ACPR). The remaining patients failed treatment and these were classified using standard WHO definitions (Table 3). Those with recurrent malaria symptoms were given a full course of rescue treatment.

**Table 3:** Classification of treatment outcome.

Classification	Number	%
**ETF**	61	64.5
**LCF**	7	7.5
**LPF**	11	11.8
**ACPR**	10	10.8
**WITH**	4	4.3

**Total analysed**	93	100

**ETF: **early treatment failure

**LCF: **late clinical failure

**LPF: **late parasitological failure

**ACPR: **adequate clinical and parasitological response

**WITH: **withdrawal.

### Sensitivity and Specificity of the qPCR assay

**Figure 1:**
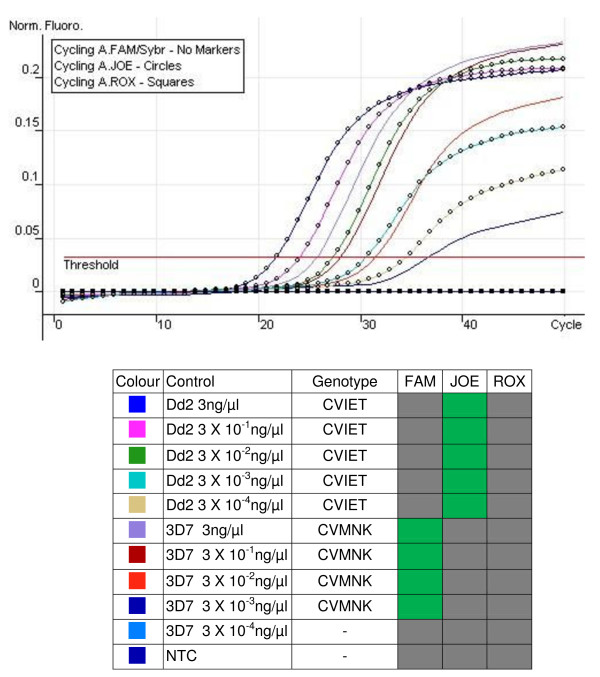
**Sensitivity of qPCR for detection of *pfcrt *alleles (CVMNK and CVIET) in *P. falciparum *DNA controls**. The sensitivity of the qPCR was tested in detecting serial dilutions of purified DNA from each control clone. Each control was prepared at a concentration of 3 ng/μl and in ten-fold dilutions to 3 × 10^-4 ^ng/μl. Green fill represents a fluorescent signal above threshold in that channel, grey fill represents no detectable signal above background. C_T _values are read from the x-axis of the plot, at the point at which the fluorescence curve crosses the threshold. Dd2: CVIET. 3D7: CVMNK. NTC: no template control.

The multiplexed qPCR assay was tested against a panel of parasite clones obtained from the MR4 repository to determine the specificity; 3D7 to represent the CVMNK, Dd2 for CVIET and 7G8 for SVMNT haplotypes, respectively. The qPCR assay detected the CVMNK at a concentration as low as 0.003 ng/μl and the CVIET and SVMNT at a concentration of 0.0003 ng/μl. Figure 1 shows the results for 3D7 (CVMNK) and Dd2 (CVIET).

### Qualitative *pfcrt *allelic discrimination

**Figure 2:**
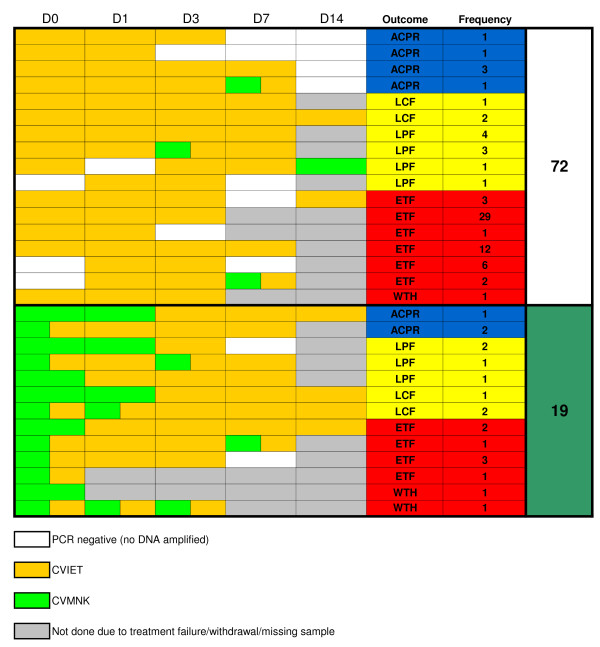
**Diagrammatical representation of *pfcrt *allele carriage among trial participants during follow-up**. Colours represent alleles carried - green for CVMNK and orange for CVIET. White cells indicate PCR was performed but no *pfcrt *DNA was detected. Shaded cells indicate no sample was taken/available. The SVMNT allele was not detected. Data collected were from single-tube assays of all available DNA samples from each participant (N = 318 data points). A number of individuals without symptoms at Day 3 and with no parasites on initial slide-reading were later found to have harboured parasites by definitive microscopy after completion of the trial. These individuals therefore contributed both Day 3 and Day 7 samples to the analysis, despite being classified ETF.

Alleles at *pfcrt *codons 72 - 76 were successfully identified in 86 of 91 available pre-treatment (day 0) samples by qPCR (5 samples failed to produce a detectable genotype). Among these, CVMNK was detected at day 0 (prior to treatment) in 19 infections (22.9%; 95% C.I. 13.7% - 32.1%), of which eleven were mixed infections that also harboured detectable CVIET. The CVIET allele, therefore, occurred in the vast majority of pre-treatment infections. Wild-type alleles became less common during follow-up, occurring in 5, 4 and 1 of the infections typed by qPCR at day 3, 7 and 14, respectively. All but the last of these were mixed infections. The SVMNT allele was not detected in any of the field samples examined in this study by either qPCR or sequencing. Carriage of *pfcrt *alleles is summarized in Figure 2. Patients displayed a number of different patterns of allele carriage during follow-up, and a total of more than 50 combinations were observed when treatment outcome classification is also taken into account (Figure 2).

The CVIET allele alone was carried throughout follow-up by 60 patients, with the majority exhibiting either early treatment failure (N = 50) or late treatment failure (N = 5). Of 19 patients carrying the CQ-sensitive allele CVMNK at enrolment, four were successfully treated, six were classed as late treatment failure, and seven as early treatment failures; two were withdrawn. The CQ-sensitive allele CVMNK was at low prevalence, and carriage of this allele prior to treatment was not significantly associated with overall treatment success (O.R. 2.32; 95% C.I. 0.332 - 12.4; *p *= 0.107 [2-sided Fisher's exact test]). However, the absence of CVMNK at enrolment was specifically associated with ETF: of the 67 (86 - 19) evaluable individuals lacking CVMNK at presentation, 53 (74.7%) suffered ETF, compared to 7 (41.2%) of the 17 evaluable individuals carrying the wild-type allele (O.R. 5.41; 95% C.I. 1.51 - 19.7; *p *= 0.002).

DNA sequencing of a 5' segment of the *pfcrt *gene was successfully performed in both forward and reverse directions for 25 of 40 randomly selected day 0 parasite DNA samples, using purified PCR products from a single round of amplification. The results were in perfect agreement with those obtained by qPCR, including one mixed infection, in which both alleles were discerned on both sequenced DNA strands.

### Quantitative analysis of *pfcrt *allele abundance during CQ treatment

**Figure 3:**
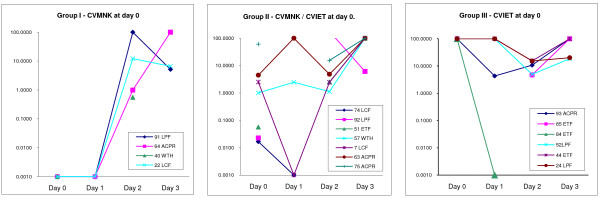
**Detailed longitudinal analysis of relative abundance of CVMNK and CVIET for 17 selected patients**. **Group I: ***P. falciparum *DNA isolated from four patients carrying only wild-type parasites at day 0. **Group II: **DNA from seven patients carrying both wild-type and CQR parasites at day 0. **Group III: **DNA from six patients carrying only CVIET parasites at day 0. Parasite DNA was analysed by qPCR to determine relative abundance of CVIET alleles compared to CVMNK alleles in each sample from day 0 to day 3. The day 0 sample from isolate 57, with similar C_T _values for both genotypes by qPCR, was selected as the comparator, and assigned an arbitrary value of 1.00. The relative abundance of the CVIET allele was then determined (see Materials and Methods). Standard WHO 14 day treatment outcomes for each individual are given in the legend of each panel: ACPR - adequate clinical and parasitological response (i.e. treatment success); WTH - withdrawn prior to day 3; ETF - early treatment failure (parasites detected on day 3, regardless of symptoms); LCF - late clinical failure (day 7 or 14, with recurrent malaria symptoms); LPF - late parasitological failure (day 7 or 14, without symptoms).

In order to examine the selective effect of CQ upon parasite genotypes, quantitative *pfcrt *allele-specific qPCR was used to measure the relative abundance of CQ-resistant genotypes in the few days immediately after treatment. This is shown in detail for the nine individuals who harboured parasites carrying the wild-type *pfcrt *allele CVMNK (alone or mixed with CVIET) at day 0, plus eight individuals carrying only CVIET parasites (Figure 3). Absolute quantification of these two genotypes would require using a standardized volume of patient blood, collected on identical filter-papers, and then extracted with similar or identical efficiency. Due to logistical problems in the field, at least two different types of filter paper had been used for blood sample collection, and DNA extraction from these two substrates may have differed in efficiency. Further, the volume of blood used in each bloodspot was not measured. Therefore, absolute quantification of parasites was not possible. To overcome this, a relative allelic quantification method was developed based on the delta-delta Ct approach used to estimate *pfmdr1 *copy number in *P. falciparum *([27]; see Methods). The results are plotted in Figure 3.

Each of the individuals with only CVMNK detected at day 0 was found to carry CVIET parasites by day 3. Two individuals initially typed as harbouring only CVIET present at day 0 were found to also have low densities of CVMNK parasites present (patients #7, #63, Figure 3B). An unexpected finding was the detection of DNA from parasites of the CVMNK haplotype at low abundance in all patients at day 2. This could not be easily explained by technical problems with the assay - all day two samples were repeated in three independent assay runs, and both positive and negative controls in each experiment gave the expected fluorescent signals. A possible explanation is that sequestered developing gametocytes and asexual parasites of the CVMNK genotype, not detectable in day 0 peripheral blood, are more quickly lysed by CQ and the DNA released reaches detectable levels on day 2 following treatment. However, an unidentified technical error or contamination affecting only these day 2 samples cannot be ruled out.

Nevertheless, at day 2 parasites of the CVIET haplotype were numerically dominant in all individuals except those who presented with CVMNK parasites only on day 0. On day 3, all individuals with parasites harboured infections completely dominated by the CVIET haplotype. Therefore, as expected, rapid and profound within-host selection against the CVMNK parasite haplotype was observed in the 72 hours following the commencement of chloroquine therapy. An important effect of this selection is to confound the identification of recrudescent parasites during follow-up. This effect is illustrated clearly by four individuals with only wild-type CVMNK parasites detected on day 0 who later failed treatment (Figure 2), and in particular by patients #22 and #91, analysed in detail in Figure 3A. The parasites detectable in peripheral blood samples have changed dramatically over the 48 hours following pre-treatment sampling, with the disappearance of the CVMNK haplotype. All these individuals failed treatment at day 7 or 14, and would be identified as "new infections" when compared against the day 0 sample only. The quantitative analysis shows that in fact the CVIET parasites appeared on day 1 or day 2 and so were certainly present prior to treatment, but were not detected until the introduction of drug to the parasite population had rapidly changed the relative abundance of sensitive and resistant parasites within each individual patient.

## Discussion

Drug resistance has been a major challenge to control programmes in sub-Saharan Africa for the past two decades, and this precipitated the policy change in Sudan from chloroquine to ACT as first-line treatment for falciparum malaria. These new drugs are now killing malaria parasites effectively, but may themselves be threatened by the development of resistance in the future, and so a thorough understanding of the selective effects of anti-malarial drugs on *P. falciparum *populations in Sudan is crucial. Here the impact of chloroquine on parasites present in the Gedaref region is reported, in the last transmission season before the ACT policy was implemented. The relative abundance of wild-type and chloroquine-resistant parasites was measured and profound and rapid within-host selection for resistant parasites in chloroquine-treated patients quantified. Further studies will measure the effects of ACT on these same parasite types in the seasons immediately following the change in treatment policy in Sudan.

In 2004, chloroquine was of almost no benefit for patients with falciparum malaria in the study area. Only 10% of treated individuals in the trial enjoyed an adequate clinical and parasitological response to treatment over 14 days, and some of these remained parasite positive by PCR at days 7 or 14 (Figure 2). Longer follow-up may have identified further clinical and parasitological failures. It was possible to identify a handful of patients harbouring drug-sensitive parasites prior to treatment, but virtually all of these were found to harbour resistant parasites later in follow-up. Detailed quantitative analysis of these individuals demonstrated that this selection for parasites of the CVIET genotype was rapid, such that there were virtually no detectable CVMNK parasites in most individuals by day 3 after treatment (Figure 3).

Particularly interesting were those individuals found to harbour only CVMNK parasites prior to treatment, but who subsequently failed treatment with CVIET parasites present. This suggests that the presence of resistant parasites among the starting population in these individuals was undetected by PCR, presumably because the presence of minority genotypes is masked by the overwhelming amplification of majority types. Chloroquine resistance brings with it a reduced fitness relative to wild-type parasites, which may mean they are out-competed by wild-type parasites in untreated infections [11]. Thus only after drug insult enhances the relative fitness of resistant parasites are they detectable in some individuals. This poses serious questions about the use of genotyping to identify recrudescent parasites during the follow-up of clinical trial participants [29], particularly in situations in which drug resistant parasites are common, and are likely to be a minority population in a proportion of infected individuals. In these circumstances, such resistant parasites may cause treatment failure later in follow-up, but will be erroneously characterized as "new infections" as their genetic fingerprint will not have been detected in the pre-treatment sample. Thus truly recrudescent, resistant parasites will not be correctly identified. In the study described here, this would have applied to four individuals, two classified as late clinical failure, and two classified as late parasitological failure (Figure 3). Although it is possible that a more sensitive genotyping technique may have detected these sub-populations at day 0, with any methodology there will always be the possibility of parasite genotypes circulating at a density below the limit of detection that will be missed by a single sampling approach. This assertion is supported by recent data from Nsobya *et al *[30], who demonstrated by expansion of parasite isolates *in vitro *that PCR genotyping of a single sample taken prior to treatment does not adequately represent all the genotypes present.

An unexpected finding was that a low abundance of parasites of the CVMNK genotype was detected in the majority of individuals at day 2 after treatment (data not shown). In all PCR-positive individuals at this point, CVIET genotypes were numerically dominant (Figure 3). A technical failing of the assay due to DNA contamination was suspected, but a repeat of the PCR analysis produced similar results. In both tests, all controls performed as expected, and no general contamination was evident in the reagents. If robust, these results could only be explained biologically if the majority of infections were a mix of CVIET and CVMNK parasites, with the latter usually undetectable at day 0. Drug effects may then have led to release of dead or damaged sequestered parasites (including gametocytes), and resulting in a small "blip" of CVMNK DNA being detected in the peripheral blood of most individuals, as well as the more resistant and abundant CVIET type. Recent studies showing the detection of *P. falciparum *DNA in the saliva of infected people may indicate the transient retention of parasite DNA in buccal macrophages [31,32], and thus phagocytes in the peripheral circulation may also provide a short-lived DNA signal from drug-killed *P. falciparum *killed in sequestered niches. This would again suggest that a single PCR amplification from peripheral blood at day 0 provides a poor representation of the parasite diversity present in an infected individual.

Apart from this question, the major weaknesses of the study were, firstly, that absolute quantification could not be performed due to variability in the volume of blood collected onto blood-spots, and to the use of two different types of filter paper. In future studies this will be rectified so that a direct comparison can be made between relative quantification methods, as used here, and absolute quantification against the international *P. falciparum *DNA standard [33]. Secondly, the assay employed shares with most PCR methods a tendency to miss those mixed genotype infections where one genotype is substantially more abundant than the other. This is because the amplified targets of the three allele-specific probes share amplification primers, and therefore the exponential amplification dynamics will lead to an over-representation of the abundant species, and a paucity of the rare species. This is demonstrated by two additional mixed infections with a low abundance of parasites with the CVMNK genotype being identified in the triplicate quantitative analysis. Both of these individuals had been typed as harbouring CVIET genotypes only in our initial qualitative analysis, which employs a conservative threshold to maximise specificity rather than sensitivity.

The current deployment of ACT for anti-malarial treatment across the African continent suggests that the acute crisis precipitated by spreading parasite resistance to chloroquine and sulphadoxine-pyrimethamine is being averted [1]. The use of ACT is likely to contribute to continuing reductions in malaria-related morbidity and mortality, such as those recently reported in The Gambia and coastal Kenya [34,35]. ACT is also threatened by the development of resistance [36,37]. Careful monitoring of the immediate quantitative effects of combination drugs on parasite burden in the first few days after treatment is therefore advisable to detect any delay in parasite clearance that may signal a reduction in drug efficacy.

## Conclusions

Evidence was presented that minor populations of drug resistant parasites are rapidly selected in the first 24 hours of antimalarial treatment *in vivo*. Thus a single pre-treatment parasite sample does not fully represent the within-host parasite population from which later recrudescent parasitaemia can arise.

## Competing interests

The authors declare that they have no competing interests.

## Authors' contributions

NBG wrote the protocol, coordinated the *in vivo *study, carried out the molecular genetic studies and quantitative PCR, and drafted the manuscript. SE and EM carried out the *in vivo *study and processed patient samples. DCW and BE conceived of the study and wrote the protocol. CJS conceived of the molecular quantitation assay, oversaw molecular studies, analysed data and wrote the revised manuscript. All authors read and approved the final manuscript.
